# Supplementary figures and images for: Differences in stress reactivity between zebrafish with alternative stress coping styles
Source: R Soc Open Sci. 2019 May 22;6(5):181797. doi: 10.1098/rsos.181797 (PMC6549991; doi:10.1098/rsos.181797)

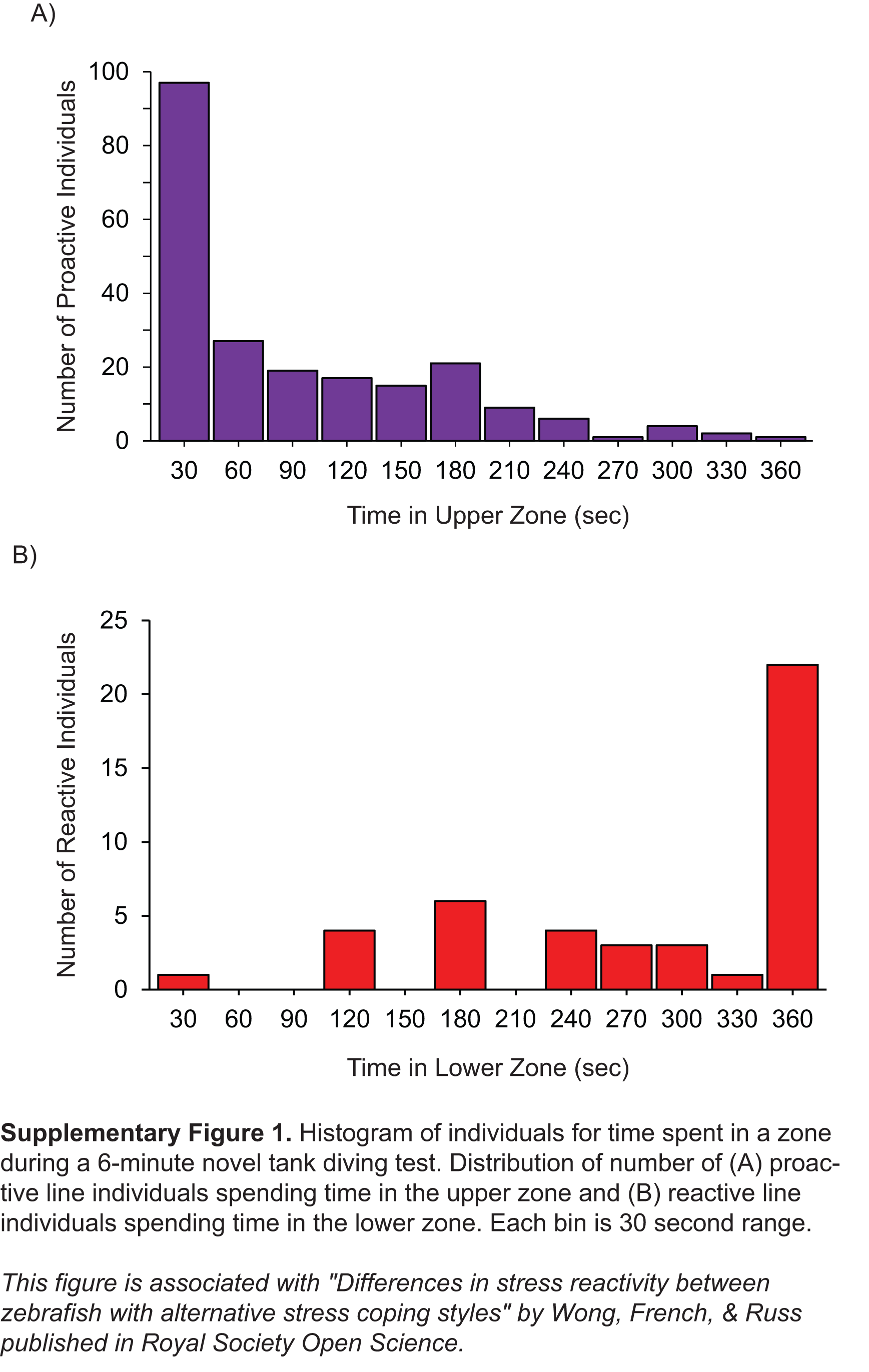

Supplement: Figure S1 [file rsos181797supp1.tif]

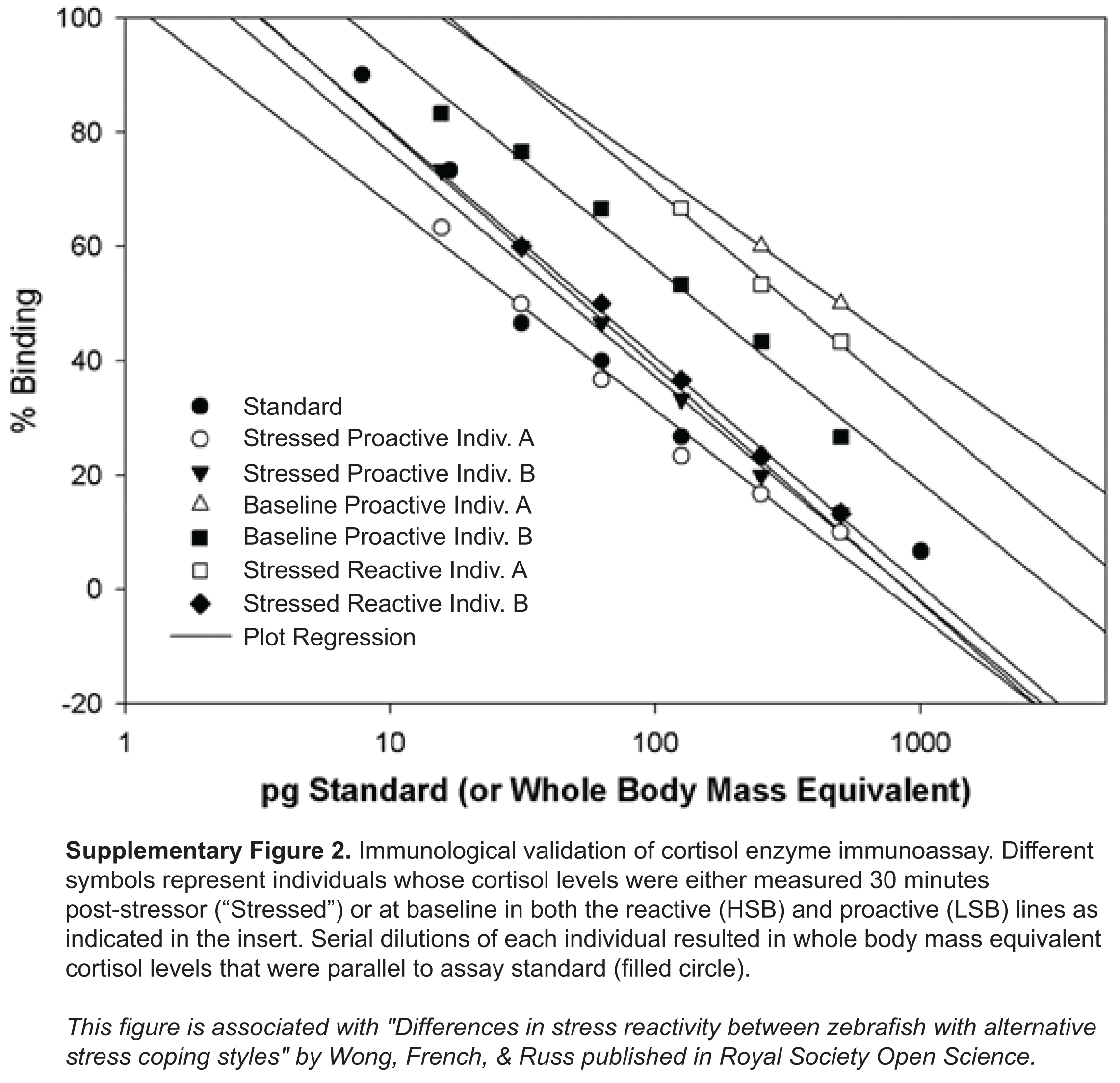

Supplement: Figure S2 [file rsos181797supp2.tif]
